# Eureka

**Published:** 1897-10

**Authors:** 


EUREKA.—The medical press confidently assert that Saranelli, of Montevide, hrs discovered separated and cultivated the true germ of yellow fever. He calls it the *bacillus ichteroides-* He found that cultivated on vegetable mold it grows and multiplies rapidly and vigorously. This affords a clue whereby it is hoped that in time the true nature and source of the poison may be learned. It is said that the great discoverer has been awarded by his government a large sum of money. If this does not turn out to be another Carmona or Domingoes Frere case, a false alarm, the hope is warranted that ere the end of the 19th century science may be enabled to protect by inoculation against yellow fever and to cure by the same means. Yellow fever being a self-limited specific disease, furnishes its own antitoxine; it is amenable, therefore, to the serum therapy and in a short time an antitoxine will be prepared in the chemical laboratory whereby yellow fever will be made to forever take a back seat.

THE occurrence of a case of yellow fever at Beaumont September 22, naturally created alarm, lest it spread. Dr. Swearingen was promptly on the spot at the first suspicious symptom, was present at the death of the boy and personally disinfected the apartments, and put into operation every resource known to science to confine the infection and prevent a spread. The sequal demonstrated the success of his efforts; no other case ocurred; a fact most creditable to him and on which the people are to be congratulated.

---

THE MEDICAL DEPARTMENT of the University of Nashville has its usual announcement in this issue. It appeared also in July, August and September. We invite especial attention to it. For years this popular school has held a conspicious place in the foremost rank of medical colleges, and has been a special favorite with Texas students. Among its *alumni* are to-day numbered some of the best known and ablest practitioners in Texas, and they are justly proud of their *Alma Mater*. Inquiries as to terms and other details, if addressed to Prof. W. G. Savage, M. D., the polite secretary, Nashville, Tenn., will receive prompt attention.

---

THE FAILURE on the part of the last Legislature to put a contingent fund at the disposal of the State Health officer is most regretable. It places him at a serious disadvantage. It will require a considerable amount of money to equip and maintain inspection stations on the Louisiana border, and the State Health officer will have to draw on his small general appropriation to meet it. This, of course, cripples him very much. Nevertheless the people in the interior may rest assured that what mortal man can do with the resources at hand Dr. Swearingen will do. He appears to have an intuitive knowledge of what to do, and does it at the right time. This faculty, joined to a thorough acquaintance with the science of sanitation and with the ways and doings of yellow fever, will enable him, we feel sure, to successfully fight the invisible enemy. His vigilance is sleepless. Should we suffer a repetition of the dreadful visitation of 1867, it will be no fault of our indefatigable health officer, but of the near sighted wisdom of those who control the finances of the State.

---

As WE GO to press (September 28th), we are able to say that there is not a case of yellow fever in Texas; but the air is full of rumors of "suspicious cases" everywhere. No one can foresee the end.

